# Role of Gonadal Steroid Hormones in the Eye: Therapeutic Implications

**DOI:** 10.3390/biom14101262

**Published:** 2024-10-07

**Authors:** Javier Valero-Ochando, Antolin Cantó, Rosa López-Pedrajas, Inmaculada Almansa, María Miranda

**Affiliations:** Department of Biomedical Sciences, Faculty of Health Sciences, Institute of Biomedical Sciences, Cardenal Herrera-CEU University, CEU Universities, 46115 Valencia, Spain; javier.valeroochando@uchceu.es (J.V.-O.); antolin.cantocatala@uchceu.es (A.C.); rosa.lopez@uchceu.es (R.L.-P.); ialmansa@uchceu.es (I.A.)

**Keywords:** gonadal steroid hormones 1, retina 2, progesterone 3, estradiol 4, testosterone 5, glaucoma 6, age related macular degeneration 7, retinitis pigmentosa 8

## Abstract

Gonadal steroid hormones are critical regulatory substances involved in various developmental and physiological processes from fetal development through adulthood. These hormones, derived from cholesterol, are synthesized primarily by the gonads, adrenal cortex, and placenta. The synthesis of these hormones involves a series of enzymatic steps starting in the mitochondria and includes enzymes such as cytochrome P450 and aromatase. Beyond their genomic actions, which involve altering gene transcription over hours, gonadal steroids also exhibit rapid, nongenomic effects through receptors located on the cell membrane. Additionally, recent research has highlighted the role of these hormones in the central nervous system (CNS). However, the interactions between gonadal steroid hormones and the retina have received limited attention, though it has been suggested that they may play a protective role in retinal diseases. This review explores the synthesis of gonadal hormones, their mechanisms of action, and their potential implications in various retinal and optic nerve diseases, such as glaucoma, age-related macular degeneration (AMD), diabetic retinopathy (DR), or retinitis pigmentosa (RP), discussing both protective and risk factors associated with hormone levels and their therapeutic potential.

Gonadal steroid hormones, such as progesterone, estradiol, and testosterone, play a vital role in the retina, influencing both developmental and physiological processes. These hormones are synthesized not only in the gonads but also within the retina itself, where they exert both genomic and nongenomic effects. Recent research has highlighted their potential protective roles in various retinal diseases, including glaucoma, age-related macular degeneration (AMD), diabetic retinopathy (DR), and retinitis pigmentosa (RP). Understanding the mechanisms through which these hormones operate and their therapeutic potential could pave the way for new treatments aimed at preserving vision and improving retinal health.

## 1. Gonadal Steroid Hormones, Synthesis

Steroid hormones represent a crucial category of regulatory substances that oversee numerous developmental and physiological functions from fetal development through adulthood [1,2]. These hormones are all derived from cholesterol, resulting in their closely related structural similarities.

Different physiological categories of steroids (gonadal steroid hormones and later mineralocorticoids and glucocorticoids) were recognized more than 70 years ago [1].

Gonadal steroid hormones, or sex hormones, were traditionally believed to be synthesized primarily by the gonads, the adrenal cortex, and the placenta [1,2,3].

These hormones play a key role not only in defining the primary and secondary sexual traits that distinguish males from females but also in regulating various body systems. Both males and females produce sex hormones, though the levels and patterns of production vary based on gender and age. The primary types of sex steroids include androgens, estrogens, and progestogens. In males, testosterone (T) is the most abundant androgen, whereas in adult females, testosterone levels are roughly 15 times lower, with androgen precursors being converted into estrogens [4,5]. Estradiol is the most common form of estrogen.

**Figure 1:**
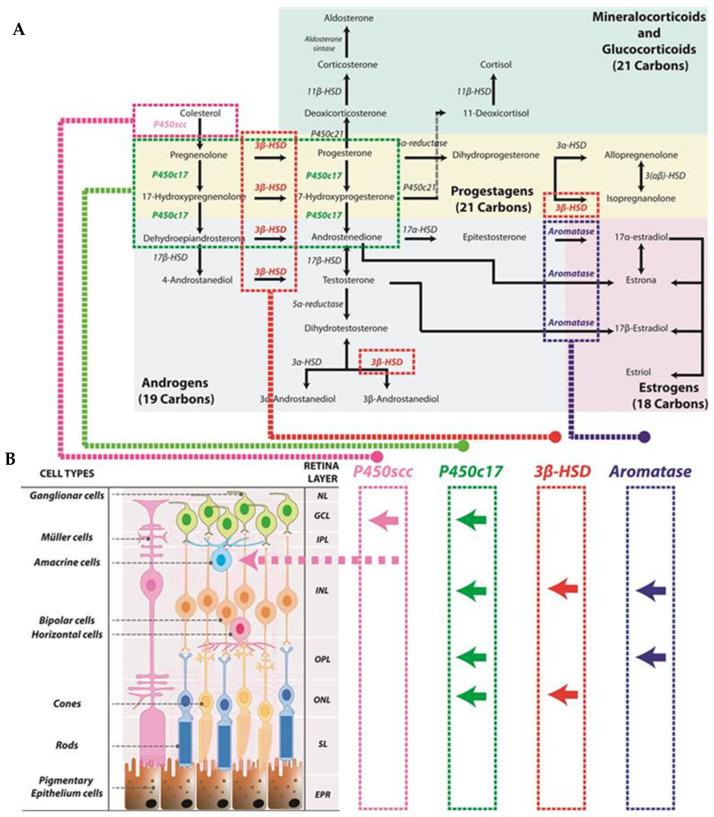
Schematic representation of steroid synthesis originated from cholesterol. This synthesis is also produced in the retina: (**A**) The diagram at the top of the figure illustrates the biosynthetic pathway of steroid hormones originating from cholesterol. Cholesterol serves as the precursor for all steroid hormones, undergoing a series of enzymatic reactions catalyzed by specific enzymes. Color coding is used throughout the figure to indicate different hormone pathways: green for mineralocorticoids and glucocorticoids; yellow for progestagens; gray for androgens; and red for estrogens. (**B**) The lower panel represents the different retinal layers and in which of these layers steroid enzymes have been found. Arrows symbolize the depicted location of the enzymes (for example, P450scc has been found in the ganglion cell layer as well as in amacrine cells). Neural layer (NL), ganglion cell layer (GCL), inner plexiform layer (IPL), inner nuclear layer (INL), outer plexiform layer (OPL), outer nuclear layer (ONL), segment layer (SL), retinal pigment epithelium (EPR).

The synthesis of gonadal hormones (Figure 1) commences within the mitochondria, where critical enzymes such as cytochrome P450 and 3β-hydroxysteroid dehydrogenase (3β-HSD) are predominantly located. Originating from cholesterol, the process involves its conversion into pregnenolone, which subsequently transitions into dehydroepiandrosterone (DHEA) and androstenedione [1]. Androstenedione acts as a precursor for testosterone, the primary androgen in humans, and for the three major estrogens: 17β-estradiol, estrone, and estriol. Estrogens are derived from androgens through the enzymatic action of aromatase [1,4,6]. More specifically, testosterone, the primary male sex hormone, is converted into 17β-estradiol and dihydrotestosterone (DHT) via the enzymes aromatase and 5-alpha-reductase, respectively [6,7]. 17β-estradiol, the main female sex hormone produced by the ovaries, can be metabolized into estrone, which can then be converted to 17α-estradiol [7]. The main site of these biochemical reactions occurs primarily in various tissues, including the gonads (testes and ovaries), adipose tissue, retina, and certain regions of the brain.

Steroid hormones have two distinct modes of action: a slower genomic effect and a faster nongenomic effect [8].

Historically, the effects of androgens and estrogens were believed to be slow and involve nuclear processes through hormone receptors found either in the cytoplasm, associated with chaperone proteins, or directly within the nucleus, such as intracellular androgen receptor (AR) and estrogen receptors (ERs) (genomic action). Typically, these intracellular hormone receptor interactions lead to the transcription of specific genes, which can take several hours to occur [3]. For example, progesterone is traditionally believed to exert its effects through the progesterone receptor (PR), a member of the nuclear steroid hormone superfamily. This receptor binds to specific progesterone response elements (PRE) located in the promoter regions of target genes, thereby regulating gene transcription [9]. Two major isoforms of the classical PR exist, PR-B and PR-A [10].

As we have just explained, the effects of gonadal steroid hormones generally take time to develop; however, some effects are rapid, occurring within minutes and leading to immediate changes. These quick responses are difficult to explain using traditional models because steroid hormones require time to bind to nuclear receptors and induce transcriptional changes. As a result, it has been proposed that the rapid, activating effects of steroid hormones, i.e., nongenomic effects, occur through various receptors located on the cell membrane [8]. Receptors for steroid hormones can initiate rapid signaling cascades through second messenger systems. This mechanism provides an additional layer of hormonal regulation that acts much faster than the genomic pathway, highlighting the complex and multifaceted roles of steroid hormones in the central nervous system. The nongenomic actions of steroid hormones often work in concert with genomic pathways, providing an intricate balance between immediate and long-term effects on brain function.

For instance, progesterone can interact with alternative receptors, which are different from the classical PR, to initiate multiple signaling pathways that influence cellular functions [8]. Some of the rapid, non-nuclear signaling pathways known to be activated by progesterone are the extracellular signal-regulated kinase (ERK) pathways, cAMP/protein kinase A (PKA) signaling, protein kinase G (PKG) signaling, calcium influx/protein kinase C (PKC) activation, and the phosphatidylinositol 3-kinase (PI3K)/Akt pathway [9]. So far, two types of cell surface proteins distinct from the classical progesterone receptors (PRs) have been identified: membrane progesterone receptors (mPRs) and progesterone membrane receptor component (PGMRC) [9]. Finally, it is interesting to note that a growing body of evidence supports the involvement of membrane-associated PRs in mediating progesterone’s effects on the brain [11]. Membrane-bound receptors for steroid hormones play a critical role in modulating neuronal activity, synaptic plasticity, and neuroprotection, often contributing to rapid alterations in mood, stress response, and cognitive functions [11].

Similarly, it has been shown that ERs are also present in extranuclear regions [9]. Additionally, research has indicated that caveolin proteins play a crucial role in estrogen-mediated signaling within hippocampal neurons, implying that ERs are situated on the plasma membrane [12]. There are other possible membrane ERs, such as G protein-coupled estrogen receptor 1 (GPER1) and the Gq-coupled membrane estrogen receptor (GqmER) [9].

## 2. Neurosteroids

Sex or gonadal hormones are not solely produced by the gonads. Increasingly, research indicates that gonadal steroid hormones are essential not only for reproductive functions but also for a range of other systems, such as the musculoskeletal, cardiovascular, and central nervous systems (CNS) [6].

In addition, it is now known that these hormones are also produced by different organs, including the CNS [13]. The concept of “neurosteroids”, introduced by Baulieu and his team in 1996, refers to steroids synthesized within the CNS that affect neurotransmission [14]. However, it is important to recognize that neurosteroids can also be generated in the peripheral nervous system.

It has been demonstrated that the essential enzyme for steroid hormone biosynthesis, known as cytochrome P-450 cholesterol side-chain cleavage enzyme (P450scc), is extensively found throughout the brain [15]. This means that neurosteroids may arrive at the CNS systemically, but they can also be locally synthesized (local neurosteroidogenesis). Indeed, enzymes necessary to synthesize steroid hormones are expressed within the nervous system. Steroidogenic enzymes that have been shown to be present in different areas of the brain include P450scc, 17α-hydroxylase, 21-hydroxylase, aromatase, 17β-hydroxysteroid dehydrogenase (17β-HSD), 5α-reductase, 3α-hydroxysteroid dehydrogenase (3α-HSD), and 3β-hydroxysteroid dehydrogenase (3β-HSD) [16]. Moreover, gonadal hormones have distinct impacts on CNS cells based on sex, as the distribution of steroid receptors varies throughout the brain between male and female nonhuman primates and rodents [17]. Testosterone levels increased in male mice around birth, with higher androgen and estrogen receptor mRNA levels observed in the hypothalamus, hippocampus, and prefrontal cortex. mRNA levels of estrogen receptor α (ERα) in the hypothalamus and hippocampus have been shown to be greater in male mice compared with female mice prior to birth. These differences likely contribute to organizing sex-specific traits in reproductive function, anxiety, stress response, and cognition [17].

## 3. Steroid Hormones in the Eye

The eye has long been regarded as a “sexually neutral” organ, meaning it was thought that there were no differences in its physiology or pathology between males and females. Today, however, it is known that the distribution of sex steroid hormones in the eye differs depending on sex and age [18]. The retina, a part of the visual system and the CNS, is now recognized as a tissue that synthesizes steroids. Within this tissue, steroid production is integrated into its neural circuits and may contribute to visual function [19].

It is well known that steroid synthesis begins with cholesterol, which is the primary precursor in steroidogenesis. In this sense, research has shown that the retina not only receives cholesterol from the bloodstream but is also capable of synthesizing it independently [20]. The balance of cholesterol within the retina depends on several interconnected mechanisms, including its new production, absorption, internal retinal movement, breakdown, and removal. The blood–retina barrier facilitates the absorption of cholesterol from the bloodstream through a mechanism involving lipoproteins and receptor mediation [21].

Disruptions in these intricate pathways can lead to various inherited or age-associated visual impairments [20]. Recently, evidence has suggested that locally synthesized steroids are essential in maintaining retinal health, particularly in conditions of oxidative or ischemic stress [19].

The predominant form of cholesterol in the retina is free, or unesterified, cholesterol. Unlike other cholesterol-producing tissues, the retina synthesizes cholesterol at a relatively slow pace [21]. However, contradictory results may be found regarding this fact, as in the retinas of hamsters, in which local biosynthesis remains the main source of cholesterol [22].

Rod cells, Müller cells, and retinal pigment epithelium (RPE) cells have the capacity to produce cholesterol [21]. Photoreceptors themselves likely produce very little cholesterol through de novo synthesis, and this is surprising, considering the high rate of membrane formation and renewal in photoreceptor outer segments, which requires a constant supply of lipids and proteins [20]. Nevertheless, research indicates that cholesterol is widely distributed throughout all layers of the neural retina.

The conversion of cholesterol to pregnenolone within the retina was first documented by Guarneri et al. in 1994 and takes place in ganglion and amacrine cells [23]. Pregnenolone is then transformed into various steroid hormones, such as progesterone, testosterone, and estradiol, through multiple enzymatic steps. Several enzymes involved in the synthesis of these hormones have been identified in different layers of the retina in male rats [24]. The enzyme P450c17 is found in the inner nuclear layer (INL), likely in neuronal-type cells, as well as in the outer plexiform layer (OPL), photoreceptors of the outer nuclear layer (ONL), and the ganglion cell layer. The enzyme 3β-HSD is present in the inner segments, ONL and INL. Additionally, aromatase has been detected in specific photoreceptor cell bodies and the outer parts of the INL and OPL [24]. Although these enzymes are present in the retina in smaller amounts than in the testes and adrenal glands, their concentrations are comparable to those in other regions of the CNS [25]. The localization of these enzymes can also be observed in Figure 1.

Regarding the mechanism of action of these hormones in the eye, several studies have localized gonadal receptors in this organ. Cascio et al. found that ER alpha is primarily located in amacrine cells and retinal ganglion cells (RGCs), while ER-beta is mostly found in the inner synaptic layer of the retina [24]. Progesterone receptors have been detected at the mRNA and/or protein levels in the retina, retinal pigment epithelium (RPE), optic nerve, and occipital cortex in both animal models and humans in both sexes [26].

In the male rat retina, the pathway of testosterone synthesis from progesterone has been characterized, indirectly indicating the formation of androstenedione via the P450c17 enzyme and its subsequent conversion to testosterone through the 17β-HSD enzyme, as reported [19]. Additionally, enzymes such as 5α-reductase types 1 and 2 and 3α-hydroxysteroid dehydrogenase (3α-HSD), which convert progesterone to allopregnanolone, have been identified in the retina [27]. The presence of 5α-reductase suggests that testosterone could be further metabolized into dihydrotestosterone (DHT) within the retina.

The identification of steroidogenic enzymes and receptors in retinal tissue underscores the functional significance of local steroid synthesis. Furthermore, steroid levels in the retina may be influenced by external factors such as light exposure and circadian rhythm.

## 4. Role of Gonadal Steroid Hormones in Eye Diseases

Several studies indicate that estrogen may play a protective role in maintaining retinal function. In this sense, differences in contrast sensitivity have been observed between women before and after menopause [28]. A decline in neuroretinal function has been observed in women who had undergone a hysterectomy during their reproductive years [29]. Additionally, a study by Olakowska et al. demonstrated that retinal ganglion cells in female Long Evans rats were more vulnerable to damage following an optic nerve crush after the rats underwent an ovariectomy procedure [30].

Though a recent work has reviewed the role of sex hormones in common ocular disorders [6], in this review, we focus only on the role of neurosteroids in the retina, as a part of the CNS, and in the optic nerve; we also focus on the use of neurosteroids as possible new treatments. We describe alterations in gonadal hormones in eye diseases such as glaucoma, age-related macular degeneration (AMD), diabetic retinopathy (DR), retinitis pigmentosa (RP), and Leber’s hereditary optic neuropathy (LHON). These conditions affect different parts of the eye, such as the retina and optic nerve, causing progressive vision loss and, in some cases, blindness. These diseases share similar pathological mechanisms, such as retinal cell degeneration, oxidative stress, and vascular dysfunction. Understanding the similarities and differences among these diseases is crucial for developing targeted therapies and preventive strategies. We also mention two other rare retinal pathologies that are acquired conditions with risk factors related to age, sex, and lifestyle: central serous chorioretinopathy (CSC) and idiopathic macular hole.

### 4.1. Glaucoma

Glaucoma is a leading cause of vision impairment and a common condition among older populations. This condition encompasses a group of eye disorders characterized by optic neuropathy, which is associated with the degeneration of RGCs, progressive loss of visual fields, and potential progression to blindness [31]. Elevated intraocular pressure (IOP) is considered the primary risk factor for developing glaucoma.

Glaucoma can be broadly classified into two main types: open-angle glaucoma (OAG) and angle-closure glaucoma. OAG is the most common form and is often asymptomatic in its early stages, leading to a gradual loss of peripheral vision and, if untreated, eventual central vision loss. Angle-closure glaucoma is less common but is considered a medical emergency due to the rapid rise in IOP and the risk of permanent vision loss if not promptly treated.

The decrease in gonadal hormones, particularly female sex hormones, has been related to a higher incidence or risk of glaucoma. Hulsman et al. reported that women who experience early menopause (before reaching the age of 45 years), whether naturally or due to irradiation therapy or bilateral ovariectomy, have a higher risk of developing OAG [32]. Hulsman et al. study was performed among the population of the Rotterdam study. Vajaranant et al. examined the risk of developing glaucoma by comparing women who had a bilateral oophorectomy with a control group of women of the same age who did not have either unilateral or bilateral oophorectomy in the Mayo Clinic Cohort Study of Oophorectomy and Aging. They concluded that women who undergo bilateral ovariectomy before the age of 43 have a significantly elevated risk of developing glaucoma, and this increased risk is not mitigated by estradiol hormone replacement therapy [33]. Premenopausal women, who have higher circulating levels of estrogen, have a lower risk of developing primary open-angle glaucoma (POAG) compared with men of the same age group. Estrogen may exert its protective effect by enhancing blood flow to the optic nerve head, reducing inflammation, and lowering oxidative stress.

It has also been demonstrated in a previous study that postmenopausal women who have reduced circulating estrogen levels experience a marked increase in glaucoma incidence and a rise in IOP of approximately 1.5–3.5 mmHg compared with premenopausal women of the same age [34]. The decline in protective estrogen and progesterone levels during menopause is one of the key factors that increase susceptibility to glaucoma in older women, often making the prevalence rates between older men and women comparable.

Being exposed to increased levels of female hormones has been linked to a decreased risk of glaucoma. Prolonged estrogen exposure, resulting from early onset of menstruation or late menopause, is linked to a reduced risk of glaucoma [35]. The use of estrogen with progestin has been associated with a reduced risk of OAG characterized by intraocular pressure > 21 mm Hg before visual field loss [36]. In 2014, Newman-Casey et al. also demonstrated that estrogen supplementation in postmenopausal women significantly lowers the risk of developing primary open-angle glaucoma [36].

Additionally, in a rat animal model of glaucoma, the administration of topical estradiol prevented the death of RGCs in a rat model of glaucoma [37].

### 4.2. Age-Related Macular Degeneration

AMD is a progressive eye condition that affects the macula. The clinical symptoms of AMD vary depending on the stage and type of the disease. Early stages of AMD may be asymptomatic, but as the disease progresses, patients may experience blurred or distorted central vision.

There are multiple risk factors for AMD, including genetic factors causing complement dysregulation, age, smoking, and sunlight exposure [38]. Age is the most significant risk factor, with the prevalence of AMD increasing dramatically in individuals over 60 years old.

Two main types of AMD have been described: dry (atrophic) AMD and wet (neovascular or exudative) AMD.

Dry AMD is the more common form, accounting for approximately 85–90% of cases [39]. It is characterized by the accumulation of drusen—yellow deposits of lipids and proteins—beneath the retina, leading to degeneration of the macular cells. As the disease progresses, it can lead to geographic atrophy, where larger areas of the retina lose their function, resulting in gradual central vision loss [40].

Wet AMD, although less common, is responsible for the majority of severe vision loss associated with AMD [40]. This form of the disease is characterized by the abnormal growth of blood vessels beneath the retina. These new blood vessels are fragile and prone to leaking, causing fluid accumulation and bleeding, which can rapidly deteriorate central vision [41].

The research and results about a possible relationship between gonadal hormones and AMD are contradictory. It has been suggested that there was a higher prevalence of AMD in women, particularly in older age groups. For example, in the Beaver Dam Eye Study, it was found that women were more likely to develop late-stage AMD compared with men, especially in the age group of 75 years and older [42]. The Eye Diseases Prevalence Research Group also reported a higher prevalence of AMD among women across various ethnic groups [43]. However, this increased risk may be associated with the fact that women have a longer life expectancy than men, leading to a greater number of women reaching the age at which AMD is most prevalent, rather than with hormonal changes [42,44].

Considering these findings, to determine whether alterations in female hormones are associated with AMD, it is essential to focus on studies that compare the risk of this disease in premenopausal and postmenopausal women or examine the effects of contraceptive use, hormone replacement therapy, and other hormonal interventions.

A recent study has investigated the risk factors associated with the development of AMD by dividing participants into two groups: menopausal and premenopausal [45]. The prevalence of AMD was compared between these two groups. The results obtained show that the incidence of AMD was not significantly different between the menopausal and premenopausal groups. However, the study found that age and diabetes mellitus were associated with an increased risk of developing AMD, regardless of menopausal status [45].

In addition, a previous study observed no relationship between late AMD or drusen larger than 125 μm, as well as the use of contraceptives, oral hormone replacement therapy, number of pregnancies, age at first childbirth, age at menarche, age at menopause, number of years of menstruation, or the cause of menopause [46]. Similar results were obtained by Abramov et al. when studying the effect of hormone therapy on the risk for age-related maculopathy in postmenopausal women [47].

Several experiments indicate that both endogenous estrogen exposure, which is influenced by factors such as the age of menarche, age of menopause, and number of pregnancies, and exogenous estrogen exposure, such as hormone replacement therapy and oral contraceptive use, may reduce the risk of developing AMD [48,49,50]. Another study has specifically shown that treatment with conjugated equine estrogens alone or in combination with progestin does not appear to influence early or late-stage AMD, but it may lower the risk of developing soft drusen or neovascular AMD [51]. Additionally, current use of hormone replacement therapy has been associated with lower odds of having large drusen, which could be predictive of advanced AMD [52].

All these studies are summarized in Table 1.

Contrary to all these studies, one study found that greater lifetime exposure to both endogenous and exogenous estrogen was associated with a higher incidence of exudative AMD [53].

The effects of gonadal hormones on AMD are complex, particularly after menopause. Steroids show both protective and potentially detrimental effects, influencing oxidative stress, inflammation, and angiogenesis (factors that are related to AMD pathology and mechanisms). Further research is necessary to understand how these hormones interact with different stages of AMD and whether hormone replacement or modulation could serve as a viable strategy for prevention or treatment.

### 4.3. Diabetic Retinopathy

DR has been historically described as a microvascular complication of diabetes mellitus that is characterized by damage to the retinal blood vessels, leading to progressive vision impairment and potentially blindness. However, over the last 15 years, numerous studies have shown that DR also affects retinal neurons [54].

DR is one of the leading causes of blindness in working-age adults worldwide and is classically classified into two main types: nonproliferative diabetic retinopathy (NPDR) and proliferative diabetic retinopathy (PDR). NPDR is the early stage of the disease and is marked by microaneurysms, retinal hemorrhages, hard exudates, and macular edema. As the disease progresses, NPDR can advance to PDR, characterized by the formation of new, fragile blood vessels (neovascularization) that grow on the surface of the retina or optic disk. These new vessels can rupture, leading to vitreous hemorrhage, retinal detachment, and severe vision loss [55].

The relationship between DR and sex has been studied in depth, although the results are contradictory.

Siddiqui et al. [56] studied the levels of estradiol in participants with and without DR among pre- and postmenopausal women with diabetes and concluded that estradiol is not related to the presence of DR. Similarly, genetic evidence based on a large sample does not support the effect of steroid hormones on DR [57].

However, other studies revealed that low serum dehydroepiandrosterone levels were significantly associated with DR in patients with type 2 diabetes mellitus [58] or that elevated serum progesterone levels are strongly linked to DR in hospitalized male patients [59].

Furthermore, what further complicates the interpretation of all these results is the fact that some other studies postulate that estrogens may exert a different action depending on the stage of diabetic retinopathy: during the initial stages, estradiol could have a beneficial effect, while during the proliferative stage it could aggravate the disease In this sense, it has been suggested that females might be partially protected against the neurodegenerative changes that occur before the onset of DR in type 2 diabetes [60] and that estrogen replacement appears to worsen the severity, potentially by inhibiting the upregulation of choroidal inducible nitric oxide synthase and stimulating macrophage activity [61]. In a mouse model of choroidal neovascularization (CNV), aged female mice had more severe CNV than age-matched males. Surprisingly, estrogen supplementation increased CNV severity, linked to decreased choroidal iNOS mRNA and increased TNF-alpha in macrophages. CNV was also more severe in iNOS-deficient mice. These results indicate that estrogen replacement may worsen CNV severity [61].

Studies exploring the role of progesterone in DR have shown promising results. One study found that progesterone reduced the osmotic swelling of retinal glial cells, which may promote cell survival in retinal explants from induced type 1 diabetic rats (Table 2) [62]. Estrogen is thought to have a protective role against the development of diabetic retinopathy, which might explain why premenopausal women with diabetes tend to have a lower prevalence and slower progression of DR compared with men of the same age group. Estrogen’s vascular protective effects are well documented; it improves endothelial function, reduces oxidative stress, and mitigates inflammation. However, after menopause, estrogen levels decline, and this protective effect diminishes. Consequently, the risk of diabetic retinopathy becomes more comparable between postmenopausal women and men.

Estrogens have also been found to exert differential effects depending on the stage of diabetic retinopathy: estradiol may have a protective role in the early stages but could potentially worsen the disease during the proliferative stage [60].

### 4.4. Retinitis Pigmentosa

The term RP describes a broad group of hereditary retinopathies, which are genetically and clinically heterogeneous. RP is a rare disease and currently has no cure [63]. It is the most common cause of hereditary blindness [64,65], with a global prevalence of 1 in 4000 individuals [66] and a total of more than one million affected people [67].

RP primarily affects the rods, which are responsible for night or low-light vision. However, once the rods have degenerated, the cones (responsible for daytime vision) also die, leading to complete blindness [66,68]. Although mutations that cause RP have been identified in different genes, the pathophysiological mechanisms that cause the death of photoreceptor cells are still unknown [69]. The first clinical signs of RP are night blindness and loss of peripheral vision (“tunnel” vision). Central vision is preserved until the advanced stages of the disease. In the later stages, affected patients show an abnormal accumulation of pigment in the peripheral retina. Symptoms usually begin in early adolescence, and severe visual dysfunction occurs around the ages of 40–50 years [70]. However, in some cases, patients experience rapid disease progression over approximately two decades. Conversely, others show a slow progression that may never cause blindness [71].

Regarding the possible protective role of one female gonadal hormone, such as progesterone in retinal degeneration, our research group has demonstrated that oral administration of 100 mg/kg of progesterone every 2 days, starting on postnatal day 7 in an animal model of RP (rd1 mice), significantly preserves the number of photoreceptors and decreases cell death [72]. Our study also highlighted the multiple benefits of progesterone, as it was able to (i) reduce the gliosis typical of this degeneration, (ii) decrease the concentration of retinal glutamate, and (iii) increase the concentration of antioxidant glutathione (GSH, reduced glutathione). Moreover, similar results were found when progesterone administration was carried out in the rd10 mouse model [73]. Our results are consistent with those found by Guarneri [74], who indicated that high levels of glutamate (as found in the retinas of rd1 and rd10 mice) lead to an alteration in the production of neurosteroids in the retina.

Other studies have shown that the administration of norgestrel in two different experimental models of RP (light-induced degeneration and rd10 mice) leads to a decrease in photoreceptor apoptosis and improves the electroretinogram [75]. Although some authors have compared these results with those of our research group, the work of both groups is very different. Synthetic progestins (such as norgestrel) are not progesterone. In fact, their chemical structure is more similar to testosterone than to progesterone. Synthetic progestins interact with progesterone receptors, but their effects can be either weaker or stronger than those of progesterone itself. Progestins also interact more strongly (compared with progesterone itself) with other receptor families, including glucocorticoid receptors and androgen receptors [76].

Finally, and similarly to the results found for progesterone mentioned previously, estradiol has also been shown to be effective in protecting photoreceptors in the light-induced retinal degeneration model. Estradiol has been shown to exert antioxidant effects, reduce inflammation, and promote cell survival, which can help protect against the degeneration of photoreceptors seen in retinitis pigmentosa [77,78,79]. We can suppose that this neuroprotective role might contribute to a slower progression of RP in women, particularly before menopause.

**Figure 2:**
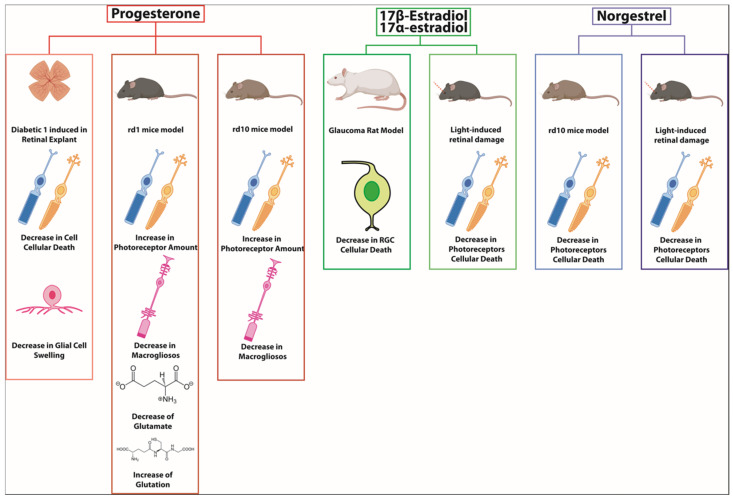
Progesterone, estradiol, and norgestrel have shown protective effects in different retinal animal models such as diabetic 1 retinopathy, rd1 mice, rd10 mice, glaucoma, or light-induced retinal damage. The action mechanism has been related to inflammation or oxidative decrease [37,72,73,74,75,76].

Different works that have administered steroid hormones to RP animal models have been summarized in Figure 2 and Table 2.

### 4.5. Other Diseases

LHON is a rare, inherited mitochondrial disorder characterized by acute or subacute vision loss due to optic nerve degeneration. It has a lower prevalence, affecting about 1 in 30,000 to 50,000 people globally. The disease is primarily caused by point mutations in mitochondrial DNA (mtDNA), with the most common mutations being ND4 (m.11778G>A), ND1 (m.3460G>A), and ND6 (m.14484T>C), which affect complex I of the mitochondrial respiratory chain, leading to impaired ATP production and increased oxidative stress [80].

LHON predominantly affects young adults, with a higher incidence in males than females, although both sexes can be affected. It is still unclear why the disease is more common in men and why it specifically targets retinal ganglion cells. Not all individuals carrying the mutation will develop the disease. Environmental factors and secondary genetic modifiers are thought to influence disease expression [81]. However, it has been demonstrated that testosterone increases apoptotic cell death and decreases mitophagy in Leber’s hereditary optic neuropathy cells [82].

Central serous chorioretinopathy (CSC) primarily affects middle-aged men and is associated with factors such as stress and corticosteroid use. The estimated incidence of CSC is about 5–10 cases per 100,000 individuals per year, with additional evidence suggesting that testosterone therapy may also increase the risk of developing CSC [83].

In contrast, idiopathic macular hole predominantly occurs in elderly women, with an incidence of approximately 8 cases per 100,000 individuals annually. This condition is more common among women than men, particularly in postmenopausal women, likely due to the sudden decrease in estrogen levels after menopause [84]. Furthermore, studies have demonstrated a potential link between tamoxifen treatment (an antiestrogen medication) and an increased risk of developing idiopathic macular holes.

## 5. Conclusions and Future Perspectives

Gonadal steroid hormones, including progesterone, estradiol, and testosterone, play a crucial role in the retina, offering potential protective effects against various retinal diseases. Their importance is evident in conditions such as glaucoma, age-related macular degeneration (AMD), diabetic retinopathy (DR), and retinitis pigmentosa (RP).

Future research should focus on understanding the molecular pathways of these hormones, conducting clinical trials to evaluate their therapeutic potential, and developing personalized treatment plans. Determining the optimal dose, timing, and tapering of hormone therapy remains crucial for successful treatment. Exploring combination therapies could also enhance therapeutic outcomes. Continued investigation into gonadal steroid hormones holds promise for developing effective treatments to preserve vision and improve the quality of life of patients.

## Figures and Tables

**Table 1:** Relation of gonadal hormone exposure with the risk of developing AMD.

Studies That Suggest a Higher Prevalence of AMD in Women	Studies That Suggest No Differences in the Risk of AMD in Premenopausal and Menopausal Women	Studies That Suggest That Estrogen Exposure Decrease Risk of AMD
Beaver Dam Eye Study: women more likely to develop AMD compared with men [42]	Incidence of AMD not different between menopausal and premenopausal groups [45]	Endogenous estrogen exposure reduces risk of AMD [48,49,50]
Eye Diseases Prevalence Research Group: higher prevalence among women [43]	No relationship between late AMD and natural exposure to estrogens and progesterone [46]	Estrogens alone or in combination with progestin lower the risk of neovascular AMD [51]
	No relationship between AMD and hormone therapy [47]	hormone replacement therapy lower risk of advanced AMD [52]

**Table 2:** Neurosteroids or gonadal hormones that have been used as possible treatments in retinal diseases. All these hormones have been used in experimental models of retinal pathologies.

Hormone	Retinal Disease and Disease Model	Observed Effect
Estradiol	Rat model of glaucoma	Prevented RGCs death
Progesterone	Retinal explants from induced type 1 diabetic rats	Reduced osmotic swelling of retinal glial cells which promote cell survival
Progesterone	Retinitis pigmentosa: rd1 mice	Preserves the number of photoreceptors; reduces gliosis; decreases glutamate, and increases GSH,
Progesterone	Retinitis pigmentosa: rd10 mice	Increases photoreceptor survival, decreases macrogliosis
Norgestrel	Light-induced degeneration and rd10 mice	Decreases in photoreceptor apoptosis and improves the electroretinogram
Estradiol	Light-induced retinal degeneration mice model	Reduces photoreceptors death
